# Isolation, Chemical Characterization and Antioxidant Activity of Pectic Polysaccharides of Fireweed (*Epilobium angustifolium* L.)

**DOI:** 10.3390/molecules26237290

**Published:** 2021-11-30

**Authors:** Sergey Popov, Vasily Smirnov, Elizaveta Kvashninova, Victor Khlopin, Fedor Vityazev, Victoria Golovchenko

**Affiliations:** Institute of Physiology of Federal Research Centre “Komi Science Centre of the Urals Branch of the Russian Academy of Sciences”, 50 Pervomaiskaya Str., 167982 Syktyvkar, Russia; smirnowich@yandex.ru (V.S.); kvashninova.e@yandex.ru (E.K.); victor1998khlopin@mail.ru (V.K.); rodefex@mail.ru (F.V.); lemnan@mail.ru (V.G.)

**Keywords:** fireweed, pectins, monosaccharide composition, structure–activity relationship, phenolics, DPPH scavenging properties, superoxide radical-scavenging activity, xylogalacturonan

## Abstract

The aim of this study was to isolate pectins with antioxidant activity from the leaves of *Epilobium angustifolium* L. Two pectins, EA-4.0 and EA-0.8, with galacturonic acid contents of 88 and 91% were isolated from the leaves of *E. angustifolium* L. by the treatment of plant raw materials with aqueous hydrochloric acid at pH 4.0 and 0.8, respectively. EA-4.0 and EA-0.8 were found to scavenge the DPPH radical in a concentration-dependent manner at 17–133 μg/mL, whereas commercial apple pectin scavenged at 0.5–2 mg/mL. The antioxidant activity of EA-4.0 was the highest and exceeded the activity of EA-0.8 and a commercial apple pectin by 2 and 39 times (IC_50_—0.050, 0.109 and 1.961 mg/mL), respectively. Pectins EA-4.0 and EA-0.8 were found to possess superoxide radical scavenging activity, with IC_50_s equal to 0.27 and 0.97 mg/mL, respectively. Correlation analysis of the composition and activity of 32 polysaccharide fractions obtained by enzyme hydrolysis and anionic exchange chromatography revealed that the antioxidant capacity of fireweed pectins is mainly due to phenolics and is partially associated with xylogalacturonan chains. The data obtained demonstrate that pectic polysaccharides appeared to be bioactive components of fireweed leaves with high antioxidant activity, which depend on pH at their extraction.

## 1. Introduction

*Epilobium angustifolium* L. (syn. *Chamaenerion angustifolium* (L.) Scop.) is a widespread, circumboreal perennial medicinal plant of the Onagraceae family. It is a native plant of many countries of the Northern hemisphere, and is commonly known as fireweed or rosebay willowherb [1]. *E. angustifolium* is often used as a domestic herbal remedy for the treatment of mouth ulcers, inflammation, dysentery, cramps, skin sores, burns, etc. Experimental studies have demonstrated that *Epilobium* extracts possess a broad range of pharmacological and therapeutic effects, including antioxidant [2], anti-proliferative [3], anti-inflammatory [4], antibacterial and other activities [5]. Biologically active compounds found in *Epilobium* species consist of flavonoids, phenolic acids, ellagitannins, β-sitosterol derivates, vitamins, fatty acids and volatile compounds [5]. Based on the importance of *E. angustifolium* in traditional medicine, and the potential for the therapeutic development of its constituents in modern medicine, it is important to investigate biologically active components of *E. angustifolium*. In this paper, we suggest that, apart from their low-weight molecular phytochemicals and polyphenols, polysaccharides may represent biologically active substances of *E. angustifolium*.

Pectin is a diverse and complex group of polysaccharides that naturally occurs in the intercellular spaces and cell walls of plants [6]. Generally, pectic polysaccharides are combined in the following structural domains: homogalacturonan (HG) composed of 1,4-linked α-d-galactopyranosyluronic acid (Gal*p*A) residues, where carboxyl groups of Gal*p*A residues may be methyl-esterified and/or, to a lesser degree, acetyl-esterified; and substituted galacturonans, which include xylogalacturonan (XGA) and rhamnogalacturonan type II. HG forms their backbone and side chains attached to the GalpA, and rhamnogalacturonan type I (RG I) has the backbone of a diglycosyl repeating unit with a strictly alternating sequence of 1,4-linked d-Gal*p*A residues and 1,2-linked l-Rha*p* residues, with the side chains formed by arabinan, galactan and/or arabinogalactan [7]. Pectins have been widely applied in the biomedical industry for their hypolipidemic, hypoglycemic, satiating, antibacterial and antitumor biological activities [8]. In particular, pectic polysaccharides from various sources (plant materials, food industry waste and modified pectins) demonstrate antioxidant activity [9,10,11,12,13]. However, despite the great antioxidant potential of exerted pectins, their active structural domain remains to be elucidated. The radical-scavenging activity of pectins can be ascribed to the presence of hydroxyls and carboxyls in the structure of these polysaccharides [14,15]. Many authors suggest that GalA, as a hydrogen donator, probably plays a key role in the scavenging of free radicals [16,17]. Hence, the HG domain may represent an active structural domain of pectin-mediating antioxidant activity [15,17]. However, some authors believe that its antioxidant activity is associated with RG-I [18], and [19] showed that antioxidant activity decreases with an increase in GalA content. Furthermore, pectins may be associated with a certain number of phenolic compounds. Feruloylation, in certain cases, occurs on the arabinose or galactose side chains of pectin polysaccharides, which might explain its considerable antioxidant potential [20].

At present, research on cell wall polysaccharides in *E. angustifolium* is scarce. Recently, an extract from *C. angustifolium* was shown to contain pectic substances [21]. However, no research on the purification, structural characterization and antioxidant activity of *Epilobium* pectins has been reported. Since different domains of the pectin macromolecule show obvious differences in the structure and level of antioxidant activity, it is important to determine with which structural domain the antioxidant activity of fireweed polysaccharide is associated.

This study is focused on the isolation, structural characterization and in vitro antioxidant activity of pectins in fresh leaves of *E. angustifolium*. Pectic fractions were then obtained using enzyme hydrolysis and anion exchange chromatography in order to explore the relationship between the structure of polysaccharide chains and their antioxidant activities.

## 2. Results and Discussion

### 2.1. Isolation and Chemical Characteristics of Fireweed Pectins

Two pectin fractions, EA-4.0 and EA-0.8, were isolated from fresh fireweed leaves with a 0.7% aqueous solution of ammonium oxalate in parallel experiments. The isolation protocol of fireweed pectins comprised three stages. The first stage was the pretreatment of the raw plant material with aqueous formaldehyde (14 h, 25 °C); this was performed in order to remove phenolic and protein compounds. The second stage was the treatment of the raw plant material by dilute hydrochloric acid in order to achieve the dissolution of insoluble pectic substances (protopectin). The third stage was the extraction of pectin substances with 0.7% aqueous ammonium oxalate at 70 °C. The difference in parallel experiments was in the treatment of fireweed leaves, which was performed at two different pH values (0.8 and 4.0). The purification of obtained extracts was performed by ultrafiltration. The treatment of raw plant materials with dilute hydrochloric acid adjusted to pH 0.8 compared to treatment at pH 4.0 resulted in higher yield pectins (Table 1). In addition, the content of protein and phenolic compounds was lower in the pectin fraction EA-0.8. The presence of phenolics in the composition of fireweed polysaccharides was expected, since phenols are linked to polysaccharide chains in many pectins [20]. For instance, total phenolic content in pectins possessing antioxidant activity was determined to be as much as 1 [22], 8 [23], 9.5 [24], 40 [25], 26–78 [26], 49–74 [27], 91 [28] and 16–161 [29] mg GAE/g. The results of the content of phenols in pectins from fireweed (EA-4.0—40 mg GAE/g; EA-0.8—27 mg GAE/g) are consistent with previously obtained data and demonstrate the effect of extraction conditions.

The polysaccharides of fractions EA-4.0 and EA-0.8 were characterized by a similar monosaccharide composition typical of pectic polysaccharides. GalA residues partially methyl etherified with methanol were major components of polysaccharides of both fractions (Table 1). The degree of methyl esterification (ca. 29%) indicated that the isolated pectins belonged to the group of low methyl-esterified pectins.

Among the pectins with antioxidant activity, only a few showed a GalA content of more than 80%. These include pectins of sunflower head [30], stems of *Equisetum sp.* [12], grapefruits [31], cladodes of *Opuntia ficus* indica [32], riang pod [26] and Chinese quince fruits [33].

The presence of regions of linear chains of 1,4-α-linked d-galactopyranosyluronic acid residues in the pectic macromolecules of EA-4.0 and EA-0.8 was detected by enzymatic digestion with 1,4-α-d-polygalacturonase with both endo- and exo- activities. Free GalA and oligosaccharides of GalA were identified by paper chromatography in the supernatant after digestion.

Based on the molar ratios obtained from the sugar compositions, the presence of structural components in the pectins was assumed. The percentages of the HG region (GalA–Rha) in EA-4.0 and EA-0.8 were 86.3 and 88.8%, respectively. These results indicated that HG regions were dominant in the fireweed pectins. The presence of HG, RG-I and XGA domains in fireweed pectin may be considered obvious, since these are common structural blocks of all pectins. However, the predominance of HG in fireweed pectin was not predictable. A large number of pectins with antioxidant activity contained less HG and more RG-I than EA: 70 and 23 [34], 60 and 20 [26], 57 and 9 [35], 55 and 42 [36], 20 and 76 [21], etc., compared to 86–89 (HG) and 8–10 (RG-I) in fireweed pectin. The Rha/GalA, (Gal + Ara)/Rha and GalA/NM ratios are used to estimate the contribution of RG-I to pectin structure, the branching degree of RG-I and the linearity of pectin, respectively. EA-4.0 and EA-0.8 exhibited low Rha/GalA (0.021 and 0.022) ratios, which confirmed that HG domains were predominant in pectins. Pectin EA-4.0 was found to have higher (Ara + Gal)/Rha and lower Gal/NM ratios than EA-0.8, which suggested the RG-I region of EA-4.0 was more highly branched. The higher ratio of 2Rha + Ara + Gal (10.2) confirmed the relative richness of EA-4.0 in RG-I compared to EA-0.8 (8.1).

EA-4.0 and EA-0.8 were found to differ twofold in the Ara/Xyl ratio (2.15 vs. 0.98), indicating differences between pectins in the structure of hemicellulosic polysaccharide domains, primarily arabinoxylans. A comparison of the Xyl/GalA ratio provides the opportunity to assess differences between pectic fractions in the presence of the XGA domain. A negligible difference was found in the Xyl/GalA ratio between fireweed pectins.

In general, the analysis of molar ratios indicates that the treatment of fireweed raw material at pH 0.8 promotes the partial degradation of isolated pectins.

Anion-exchange chromatography on DEAE cellulose (OH^−^) revealed that the pectin fractions EA-4.0 and EA-0.8 were characterized by significant heterogeneity. Six fractions were obtained by separating EA-4.0 and EA-0.8 (Table 2). The fraction eluted with 0.2 M NaCl has the highest yield and the fractions eluted with 0.3 M NaCl and 0.2 M NaOH have the lowest yield when fractionated with both EA-4.0 and EA-0.8.

The monosaccharide compositions of the polysaccharides in these fractions obtained on DEAE-cellulose did not differ markedly and were similar to each other and to the parent polysaccharides; residues of GalA predominated in all fractions (Table 2). All pectin fractions were characterized by the absence of protein, indicating the absence of covalent bonds between the protein and polysaccharide components of the EA-4.0 and EA-0.8. HPSEC analysis of the corresponding fractions obtained by fractionation of EA-4.0 and EA-0.8 on DEAE-cellulose confirmed a higher degradation of pectins extracted at a pH of 0.8 due to Mw; samples obtained during fractionation of EA-0.8 were lower than during fractionation of EA-4.0. However, the treatment of raw material at pH 0.8 led to a lower presence of arabinoxylans in pectin fractions.

The enzymatic treatment of EA-4.0 and EA-0.8 by polygalacturonase was used for hydrolysis of galacturonanic regions. Enzymatic hydrolysis have been shown to remove about 80% of the GalA residues of pectins [37]. Enzymatic hydrolysis is the preferred strategy for hydrolyzing pectin: The HG backbone of the pectin is resistant to acid hydrolysis, and acid treatments that are sufficiently strong to hydrolyze this backbone also degrade a significant proportion of the sugars that are released [38]. As a result of twofold exhaustive treatment of EA-4.0 and EA-0.8, oligo- and polysaccharides with different molecular and structure characteristics were obtained (Table 3 and Table 4). The polysaccharide fractions obtained by precipitation with a fourfold volume of 95% ethanol (final concentration 79%) were mainly at the yielding stage. They were resistant to enzymes and were characterized by a higher degree of methyl esterification of Gal*p*A residues (DM 37.6 and 33.8%) than parent EA-4.0 and EA-0.8, respectively. The oligosaccharide fraction EtOH-1 was obtained by precipitation with a fivefold volume of 95% ethanol (final concentration 79%) from EA-4.0. The oligosaccharide fraction EtOH-2 was obtained by precipitation with a fivefold volume of 95% ethanol (final concentration 81%) from EA-0.8. Other oligosaccharide fractions were obtained in low yield by precipitation with ethanol with a higher concentration (only from EA-4.0).

Residues of enzymatic hydrolyses were fractionated by anion-exchange chromatography on DEAE-cellulose (OH^−^). Fractions characterized by a high content of residues of neutral monosaccharides and longer regions of rhamnogalacturonan-I were obtained by elution with 0.01 M NaCl and 0.2 M NaOH. Fractions eluted with 0.1, 0.2 and 0.3 M NaCl were similar to the parent. It was found that all fractions obtained by anion-exchange chromatography included non-methyl-esterified GalA residues. This is because the separation of pectins on the anion-exchange column with DEAE cellulose (OH^−^) caused the saponification of methyl esters.

### 2.2. DPPH Radical-Scavenging Activity

**Figure 1:**
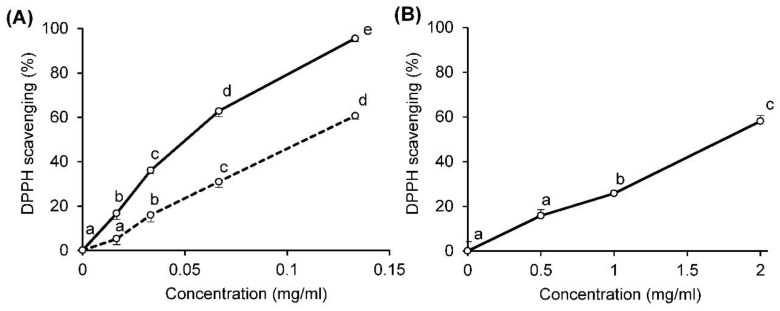
The DPPH scavenging activity of pectins EA-4.0 and EA-0.8 (**A**) in comparison with commercial apple pectin (**B**). Solid and doted lines in Figure 1A show pectins EA-4.0 and EA-0.8, respectively. Data are presented as the mean ± SD of three independent experiments. Different lowercase letters (a, b, c, d, e) for the same sample at different concentrations indicate significant differences (*p* < 0.05, LSD test).

EA-4.0 and EA-0.8 were found to scavenge the DPPH radical in a concentration-dependent manner at 17–133 μg/mL (Figure 1A), whereas commercial apple pectin scavenged at 0.5–2 mg/mL (Figure 1B). The antioxidant activity of EA-4.0 was the highest and exceeded the activity of EA-0.8 and a commercial sample by 2 and 39 times (according to IC_50_), respectively (Table 5). It was found that fireweed pectins, not washed and washed with ethanol, demonstrated the same activity (IC_50_: EA-4.0, 0.051 vs. 0.050 mg/mL, *p* = 1.00; EA-0.8, 0.103 vs. 0.109 mg/mL, *p* = 0.66). Thus, fireweed pectins possess a high antioxidant activity, which depends on the pH of the extraction.

Determination of DPPH scavenging activity is carried out by different laboratories at different ratios of pectin and DPPH radical, which makes it difficult to compare the antioxidant activity of different pectins. In a number of works, the same ratio of pectin and DPPH radical was used as in our study, where the ratio of pectin to radical was 1:1 (*v/v*) at a radical concentration of 0.2–0.3 mM. A comparison of data from these studies indicates that the activity of fireweed pectins (IC_50_—0.05 and 0.109 mg/mL) seems to be comparable to that of polysaccharides from *Lonicera japonica* Thunb (0.01–2.00 mg/mL) [39], mulberry fruits (0.17–0.32 mg/mL) [40] and *Acanthopanax senticosus* leaves (~0.3 mg/mL) [41]. It should be noted that some other pectins demonstrate DPPH scavenging activity at much higher concentrations. These include pectins from hawthorn wine pomace (1.71 and 2.10 mg/mL) [42], dandelion roots (>2.5 mg/mL) [43], apple pomace (3.02–5.24 mg/mL) [19], Cassia seed (4.41 and 5.83 mg/mL) [10] and quinoa (5.22–15.22 mg/mL) [9].

**Figure 2:**
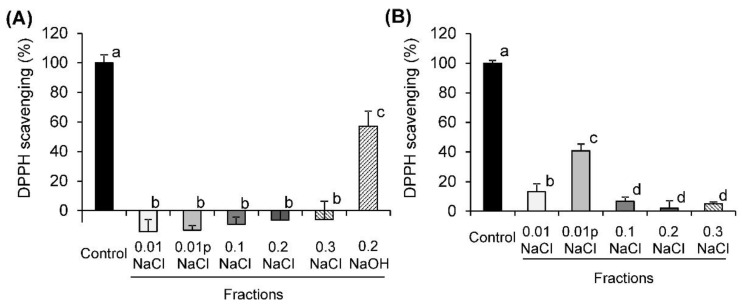
The DPPH scavenging activity of fractions of pectins EA-4.0 (**A**) and EA-0.8 (**B**) obtained by their fractionation on DEAE-cellulose column in comparison with the original pectin (control). Data are presented as the mean ± SD of three independent experiments. Different lowercase letters (a, b, c, d) indicate significant differences (*p* < 0.05, LSD test).

The antioxidant activity of the fractions obtained by the elution of EA-4.0 and EA-0.8 pectins on DEAE-cellulose was compared with the activity of the parent pectin (control). The concentration of the fractions corresponds to the half-inhibiting dose of the pectin (50 and 109 μg/mL for EA-4.0 and EA-0.8, respectively) in order to detect both lower and higher activity of the fractions. It was found that the fractions from EA-4.0 eluted with NaCl did not scavenge the DPPH radical (Figure 2). The fraction eluted with NaOH showed 57% of the activity of control. Two fractions from EA-0.8 eluted with 0.01 and 0.01p NaCl scavenged 13 and 41% of the radical of control, respectively. The NaOH fraction from EA-0.8 had a low yield compared to the rest of the fractions, so its activity was not determined.

**Figure 3:**
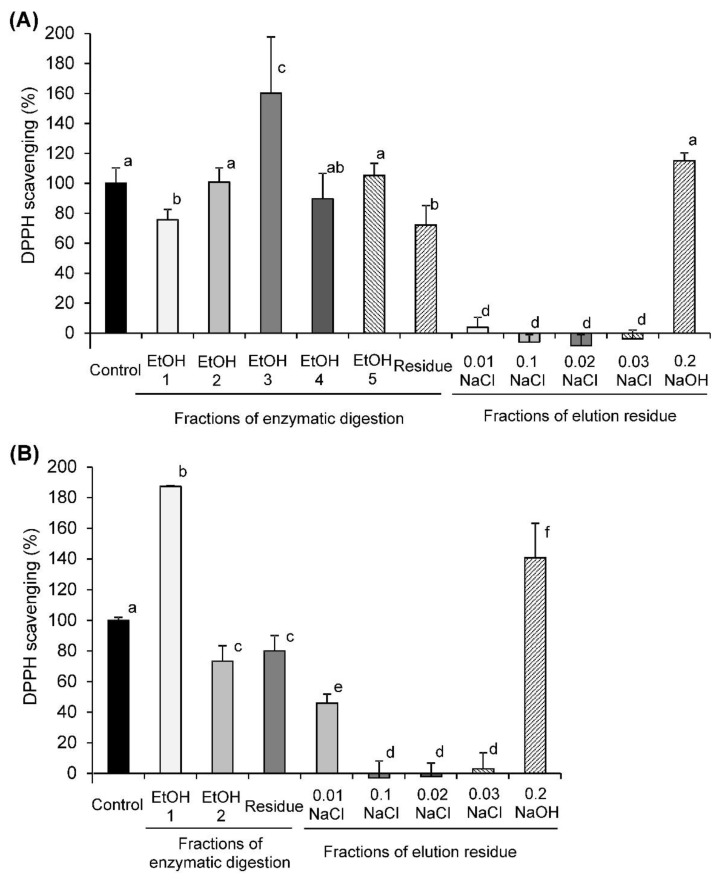
The DPPH scavenging activity of fractions obtained by elution of pectins EA-4.0 (**A**) and EA-0.8 (**B**) by the enzymatic digestion and by fractionation of residue on DEAE-cellulose column in comparison with the original pectin (control). Data are presented as the mean ± SD of three independent experiments. Different lowercase letters (a, b, c, d, e, f) indicate significant differences (*p* < 0.05, LSD test).

All fractions obtained by enzymatic hydrolysis were found to scavenge the DPPH radical with efficiencies around 72–187% of the original pectin (Figure 3). Fraction 3 from EA-4.0 and fraction 1 from EA-0.8 precipitated by 83 and 79% ethanol were the most active (160 and 187% of control). Other fractions and residues obtained by enzymatic hydrolysis of EA-4.0 and EA-0.8 showed similar or less activity (72–105%) than the control. It was found that the fractions obtained after elution of the residues on DEAE-cellulose scavenged the DPPH radical with the same pattern as the control (Figure 2 and Figure 3). Therefore, the results reveal that enzymatic hydrolysis leads to the release of lower molecular weight fragments of pectins that scavenge the DPPH radical.

The relationship between the DPPH scavenging activity and the chemical characteristics of fireweed pectins and their fractions has also been investigated. The content of the GalA, the HG domain (GalA/NM and GalA–Rha ratios) and arabinoxylans (Ara/Xyl) indicators were shown to negatively correlate with DPPH scavenging activity (Table 6).

Antioxidant activity of the samples was found to positively correlate with the content of proteins, phenolics, xylose, glucose, arabinose, the (Ara + Gal)/Rha and the Xyl/GalA ratios. To identify the characteristic(s) most responsible for the significantly higher antioxidant activity of the pectins, a multiple regression analysis was performed. Only the content of phenolics and the Xyl/GalA ratios were included in the regression model (Table 7). Four independent variables (content of proteins, Xyl, GalA and Glc) were removed from the regression due to multicollinearity, and another five (content of arabinose, the Ara/Xyl, (Ara + Gal)/Rha, GalA/NM and GalA–Rha ratios) were removed due to their lower significance (according to *p* value). It was found that linear regression resulted in a good model for prediction (R^2^ = 0.90, *p* = 0.000). Significant factors contributing to activity were the content of phenolics and the Xyl/GalA ratio (β = 0.85 and 0.20), respectively.

Correlation analysis revealed that the content of phenolics and xylogalacturonan (the Xyl/GalA ratio) determined the antioxidant activity of fireweed pectins, which partly agrees with the data of other authors. In particular, Wikiera et al. [44] found a close relationship between the binding of the DPPH radical and the content of phenolics, fucose and proteins (R^2^ = 0.94, 0.80 and 0.80, respectively) in apple pectins. In [45] and [46], it was shown that antioxidant activity towards the DPPH radical was positively related to the content of arabinose and negatively related to the content of uronic acids (galacturonic and glucuronic) in polysaccharides of various structures. However, these authors did not include phenolics in their analysis, which explains the lower efficiency of their regression models (R^2^ = 0.28–0.46 [45], 0.70 [47], 0.81 [46], compared with the present study (R^2^ = 0.90). The content of phenolics in fireweed pectin was determined to be more strongly associated with the content of glucose (R^2^ = 0.76, *p* = 0.000) and xylose (R^2^ = 0.57, *p* = 0.004) than arabinose and galactose (R^2^ = 0.19 and 0.12, *p* > 0.1).

There are very few studies showing the antioxidant activity of XGA in pectins. Pectic polysaccharides possessing free radical-scavenging activity were recently extracted from *Averrhoa bilmbi*. The higher molar ratio of GalA to xylose indicated that this pectin appeared to be a xylogalacturonan [48]. Our results also indicate the antioxidant activity of the XGA chains.

### 2.3. Superoxide Radical-Scavenging Activity

**Figure 4:**
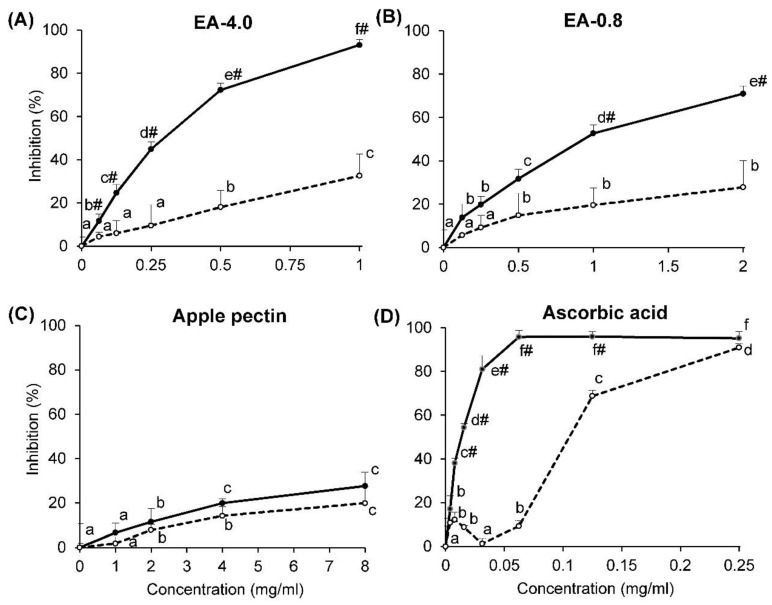
The inhibition reduction in ferricytochrome *c* (solid line) and activity of xanthine oxidase (dotted line) by pectins EA-4.0 (**A**) and EA-0.8 (**B**) in comparison with commercial apple pectin (**C**) and ascorbic acid (**D**). Data are presented as the mean ± SD of three independent experiments. Different lowercase letters (a, b, c, d, e, f) for the same reaction at different concentrations indicate significant differences (*p* < 0.05, LSD test). #—the differences are significant compared with the xanthine oxidase inhibition (*t*-test).

A xanthine/xanthine oxidase system was used to determine the rate of generation of superoxide radicals. A decreased production of superoxides was measured using the ferricytochrome *c* reduction assay, and the inhibition of xanthine oxidase was measured in terms of the production of uric acid. It was found that pectins from fireweed inhibited the rate of ferricytochrome *c* reduction and the production of uric acid, depending on their concentrations (Figure 4A,B). Inhibition of the first reaction was, on average, 29 and 42% more than the second for EA-4.0 and EA-0.8, respectively. Therefore, the inhibition of the production of superoxide radicals by fireweed pectins was due to the inhibition of xanthine oxidase and the scavenging of superoxide radicals. Ascorbic acid, used as a positive control, had the same effect (Figure 4D). Commercial apple pectin inhibited both reactions to the same extent (<30%), which indicates that it does not scavenge the superoxide radical (Figure 4C). It was calculated that the IC_50_ for the inhibition of cytochrome *c* reduction is equal to 0.27, 0.97, >8 and 0.013 mg/mL for EA-4.0, EA-0.8, commercial sample and ascorbic acid, respectively. Thus, fireweed pectins scavenged the superoxide radical to a much greater extent than the commercial apple pectin.

## 3. Materials and Methods

### 3.1. Materials

The fireweed leaves were collected in July 2017 from plants grown in Komi Republic (Latitude: 61′14″ S; Longitude: 50′24″ E), Russia. Plant material was botanically identified by Dr. Nina N. Shergina from Syktyvkar State University, Syktyvkar, Russia.

Reagents, including bovine serum albumin, catalase, cytochrome *c*, DEAE-cellulose, 3,5-dimethylphenol, 1,1-diphenyl-2-picrylhydrazyl radical (DPPH), the Folin and Ciocalteu’s phenol reagent, myo-inositol, 1,4-α-d-polygalacturonase, rhamnose (Rha), arabinose (Ara), galactose (Gal), mannose (Man), xylose (Xyl), glucose (Glc), galacturonic acid (GalA), sodium borohydride, sodium chloride, superoxide dismutase, xanthine and xanthine oxidase were purchased from Sigma-Aldrich (St. Louis, MO, USA). The pullulan standards were purchased from Fluka (Steinheim, Germany) and PSS Polymer Standards Service GmbH (Mainz, Germany). Gallic acid was purchased from MP Biomedicals (Solon, OH, USA).

### 3.2. Isolation of Pectins

Isolation was carried out as described in an earlier method [49], with some modifications. The fresh plant material (6.4 kg) was preliminarily soaked in solution of 1.0% (*v*/*v*) aqueous formaldehyde (40 L) for 14 h at 25 °C, followed by filtration and washing in water. The residual raw plant material was separated into two equal parts and each part was treated with aqueous hydrochloric acid (30 L) with a different pH (one—4.0 and second—0.8) for 3 h at 50 °C, followed by filtration. Then, all residues of raw plant material were extracted with 0.7% (*w*/*v*) aqueous ammonium oxalate (30 L) for 6 h at 70 °C, which resulted in the isolation of the extract of pectin polysaccharides. The extracts were successively filtered using filter cloth and streaming centrifuging (Avanti J-25I, Beckman Coulter, Unterschleißheim, Germany) at 10,000 rpm for 4–6 h at 4 °C. Then, the supernatants were dialyzed and concentrated by ultrafiltration. During ultrafiltration, extracts were stored in a 69 L feed tank and cycled via a pump. The four membranes (PS 302 146690, filter cartridge module Vladirsart, Vladimir, Russia) had a general surface area of 2.8 m^2^ and a cut-off molecular weight of 300 kDa. Ultrafiltration was conducted under 1–4 bar at 25–40 °C. Retentate was collected in the tank, concentrated to 20 L and treated with aqueous HCl to pH 4.0–5.0. The solution pH was read on a pH meter. Then, filtration was conducted in constant volume diafiltration mode in order to maintain a high desalination efficiency. Ultrafiltration was carried out for a negative reaction on carbohydrates filtrate (the reaction with phenol in the presence of concentric sulfuric acid) [50] on the chloride and oxalate ions (the qualitative reaction with silver nitrate), after which the ultrafiltration was stopped and the retentate was concentrated to 5 L. The resulting purified solutions of pectin polysaccharides were collected, concentrated using a Laborota 4002 rotary evaporator (Heidolph, Schwabach, Germany) under reduced pressure at 40–45 °C and lyophilized from a frozen state using a VirTis lyophilizer (VirTis, Tillson, NY, USA) under a constant vacuum of <100 mTorr at −65 °C. The samples were periodically removed and weighed to assure a constant mass after 6 h and they were dried further if the sample mass changed by more than 5% during the previous 2 h of lyophilization. The two pectin polysaccharides obtained were designated as EA-4.0 (11.0 g, yield 0.89%) and EA-0.8 (16.9 g, yield 1.29%). The yields of fractions obtained are expressed in % (*w*/*w*) of mass of dry plant material and are presented in Table 1. Results are presented as mean values of analytical triplicates, and the reproducibility of the results is expressed as pooled standard deviations (Pooled SD). Pooled SD were calculated for each series of replicates using the sum of individual variances weighted by the individual degrees of freedom [51].

### 3.3. General Analytical Methods

Absorbance was measured using an Ultrospec 3000 spectrophotometer (Pharmacia Biotech, Cambridge, UK). The solutions were concentrated with a Laborota 4002 rotary evaporator (Heidolph, Schwabach, Germany) under reduced pressure at 40 °C. The samples were centrifuged at 11,000 rpm at 4 °C for 10–20 min on a Sigma 6 K 15 centrifuge with rotor N 12.256 (Sigma Laborzentrifugen GmbH, Osterode am Harz, Germany), and were then lyophilized using a VirTis lyophilizer (VirTis, Tillson, NY, USA) under a constant vacuum of <10 mTorr at −65 °C.

The content of uronic acids was determined by reaction of the sample with 3,5-dimethylphenol in the presence of concentrated H_2_SO_4_ using the calibration plot for d-Gal*p*A. Photocolorimetry was then carried out at two wavelengths, 400 and 450 nm [52].

Quantitative determination of protein was calculated using the Bradford method using the calibration curve plot for bovine serum albumin. Photocolorimetry for this assay was carried out at 595 nm [53].

The quantitative determination of phenolics was performed with the Folin–Ciocalteu reagent using gallic acid as a standard [54]. The results were expressed as mg of gallic acid equivalents (GAE) per gram of pectin.

The monosaccharide composition of polysaccharides was determined by gas–liquid chromatography (GLC), as described earlier [55].

The content of methoxyl groups was determined by a previously described method [56] and by using the calibration plot for methanol, spectrophotometrically carried out at 412 nm. The degree of methylation was calculated as the molar ratio between methanol and uronic acids. Each experiment was run in triplicate.

The relative molar mass distribution (RMM) of the polysaccharide samples was determined by size-exclusion chromatography (HPSEC) of polysaccharides with high-performance liquid chromatography (HPLC). The chromatographic system consisted of an LC-20AD pump, a DGU-20A3 degasser, a CTO-10AS column thermostat, an RID-10A refractometric detector (all Shimadzu, Kyoto, Japan), a PPS SUPREMA 3000A 10 µm column (8.0 mm × 30 cm) and a PPS SUPREMA 10 µm precolumn (8.0 × 50 mm). The HPSEC experiments were performed at 40 °C with a flow rate of 0.4 mL/min. The column was equilibrated with 0.15 M NaCl containing 0.02% NaN_3_ as a preservative, and elution was carried out with the same solution. Deionized water supplied by the Simplicity 185 Millipore water purification system (Millipore SAS, Molsheim, France) was used to prepare the eluents and samples. Pullulans (Mw 0.342 (RT 30.649 min), 1.3 (RT 30.404 min), 6.2 (RT 29.068 min), 10.0 (RT 28.735 min), 21.7 (RT 27.842 min), 48.8 (RT 26.589 min), 113.0 (RT 25.068 min), 200.0 (RT 23.782 min), 366.0 (RT 22.190 min), 805.0 (RT 20.336 min) × 103 g/mol (PSS Polymer Standards Service GmbH, Mainz, Germany), 5.9 (RT 29.162 min), 11.8 (RT 28.632 min), 22.8 (RT 27.739 min), 47.3 (RT 26.704 min), 112.0 (RT 25.184 min), 212.0 (RT 23.673 min) and 404.0 (RT 21.993 min) × 103 g/mol (Fluka, Mainz, Germany)) were used as standards, resulting in a loglinear relationship between relative molar mass distribution and elution time. As noted earlier, the resulting standard curve allowed for only a relative estimation of molar mass distribution of the pectic polysaccharides due to the slight differences in hydrodynamic volumes expected for pullulan and pectic polysaccharides with the same molar mass distributions [57]. Number (Mn) and weight (Mw) of average relative molar masses, as well as polydispersity indices (PDI), were calculated by the LCsolution GPC program (LCsolution, Kyoto, Japan, version 1.24 SP1). The samples and standards were injected twice.

### 3.4. Anion-Exchange Chromatography

Pectin polysaccharides (100 mg) were dissolved in 5 mL of 0.01 M NaCl, and the solution was applied to a DEAE-cellulose (OH^−^) column (2.5 cm × 40 cm). The column was stepwise eluted with distilled water and 0.01, 0.1, 0.2, 0.3, 0.5 M NaCl and 0.2 M NaOH solution (400 mL of each eluent) at a flow rate of 1.0 mL/min. The fractions were collected at 10 min intervals using a low-pressure system from Pharmacia Biotech (Uppsala, Sweden) with a FRAC-100 fraction collector, a P-50 pump and a Uvicord SII. The carbohydrate content in each tube was determined by the phenol–sulfuric acid method [50]. Six pectin fractions were obtained at each separated sample: two eluted with 0.01 M NaCl (one of which was pigmented), and one by one when eluted with solutions with 0.1, 0.2, 0.3 M NaCl and with 0.2 M NaOH. The yields and chemical characteristics of pectin fractions are presented in Table 2, Table 3 and Table 4.

### 3.5. Enzymatic Digestion

Each pectin fraction, EA-4.0 and EA-0.8 (1 g), was dissolved in water (100 mL); then, an aqueous solution (1 mL) of 1,4-α-d-polygalacturonase (15 mg, endo- and exo-activities, 690 units/g, EC 3.2.1.15) was added and the mixture was incubated in a shaking incubator at 25 °C. The digestion was controlled according to [58], each half an h to estimate the reducing sugar quantities. After stopping the growth in the amount of reducing sugars, an aqueous solution (1 mL) of 1,4-α-d-polygalacturonase (10 mg) was added again. After stopping the growth in the amount of reducing sugars, fermentation was inactivated by boiling at 100 °C and removed by centrifugation.

To separate the components of the hydrolysates, fractional precipitation of polysaccharides with ethanol of increasing concentrations was used. The supernatant was concentrated and the pectins were first precipitated with a fourfold volume of 95% ethanol (final concentration, 76%). The precipitate was separated by centrifugation, washed twice with 95% ethanol, dissolved in water, frozen and then lyophilized, resulting in the residue of enzymatic hydrolyze.

The ethanol supernatant obtained after precipitation of enzymatic hydrolyze of residue was analyzed by paper chromatography for the presence of d-galacturonic acid, and then concentrated using a rotary vacuum evaporator to a minimum volume. The precipitation procedure was repeated, the supernatants were concentrated and the pectins were precipitated in stages with fourfold (final concentration 76%), fivefold (final concentration 79%), sixfold (final concentration 81%), sevenfold (final concentration 83%), eightfold (final concentration 84%) and tenfold (final concentration 87%) volumes of 95% ethanol. As result, five pectin fractions were obtained from the EA-4.0 enzymatic hydrolysate and two pectin fractions were obtained from the EA-0.8 enzymatic hydrolysate. The yields and chemical characteristics of pectin fractions are presented in Table 3 and Table 4.

### 3.6. Antioxidant Activity

The DPPH radical-scavenging activity of the pectins was assayed according to [59], with a slight modification. We added 0.6 mL 0.2 mM DPPH in ethanol to 0.6 mL pectin solution (0.3–4 mg/mL) in water, which was shaken. After incubating at 25 °C for 1 h, the absorbance of the sample was measured at 517 nm. The scavenging activity of the pectins was measured at seven different concentrations and the half-maximal inhibitory concentration (IC_50_, mg/mL) values were calculated based on a polynomial regression curve. The scavenging activity of the fractions was expressed as a percent of inhibition in comparison with the activity of the original pectin (at a concentration equal to IC_50_ of pectins).

The superoxide radical-scavenging activity of the pectins was measured by continuously measuring ferricytochrome *c* reduction, as has been reported previously [60]. The reaction mixture consisted of a 300 μL reaction mixture comprising PBS (0.1 M, pH 7.8), ethylenediaminetetraacetic acid (0.1 mM), xanthine oxidase (27 × 10^−3^ U/mL), xanthine (50 μM) and ferricytochrome *c* (20 μM). The assay was initiated by adding the enzyme to the reaction mixture, with or without a sample. Ascorbic acid was used as positive control. The assay mixture was incubated at 25 °C for 5 min, and the absorbance was recorded each 20 s. All data obtained from the enzyme kinetic assays were recorded in matched quartz plates (Hellma, Mülheim, Germany) and were plotted using KC4 software on a PowerWave 200 microplate scanning spectrophotometer (Bio-Tek Instruments, Winooski, VT, USA). The IC_50_ values of the samples were calculated based on a polynomial regression curve. The inhibition of xanthine oxidase activity was observed using the method described above. The reaction mixture consisted of the same components, except for ferricytochrome *c*, and the absorbance was measured at 290 nm.

### 3.7. Statistical Analysis

The significance of difference among the means in determining antioxidant activity was estimated with a *t*-test, one-way ANOVA and Fisher’s least significant difference (LSD) post hoc test at *p* < 0.05. The relationship between the chemical characteristics and activity of polysaccharide fractions (*n* = 32) was evaluated by calculation of the Pearson correlation coefficients and multiple linear regression analysis. All calculations were performed using the statistical package Statistica 10.0 (StatSoft, Inc., Tulsa, OK, USA). The data shown were expressed as the means ± SD of three independent experiments.

## 4. Conclusions

In conclusion, our study shows that polysaccharides isolated from fresh leaves of *E. angustifolium* represent pectins with predominant HG chains, branched RG-I and XG domains. Fireweed pectins were shown to possess antioxidant activity, which itself depends on structural features. The antioxidant activity of pectin isolated at pH 4.0 is two times higher than that of pectin isolated at pH 0.8, indicating the effect of the extraction conditions. Correlation analysis of the composition and activity of polysaccharide fractions obtained by enzyme hydrolysis revealed that the antioxidant capacity of fireweed pectins is mainly due to phenolics and is partially associated with xylogalacturonan chains.

## Figures and Tables

**Table 1:** Chemical characteristics of the polysaccharide fractions extracted from *E. angustifolium*.

Characteristic	EA-4.0	EA-0.8
Yield, % ^1^	0.89	1.29
Uronic acid, % ^2^	88.2	90.8
DM ^2^	29.4	28.5
Protein, % ^3^	2.6	0.3
Phenolic, % ^3^	4.0	2.7
Rha, % ^2^	1.9	1.9
Ara, % ^2^	3.3	1.7
Xyl, % ^2^	1.5	1.7
Man, % ^2^	0.5	0.6
Glc, % ^2^	1.5	0.8
Gal, % ^2^	3.1	2.6
Total sugar, % ^3^	86.6	71.8
GalA/NM	7.50	9.82
Rha/GalA	0.022	0.021
Ara + Gal/Rha	3.29	2.22
GalA–Rha	86.31	88.84
2Rha + Ara + Gal	10.16	8.10
Ara/Xyl	2.15	0.98
Xyl/GalA	0.017	0.019

^1^ per weight of dry wt. ^2^ mol%. ^3^ wt%. Pooled SD: pooled standard deviation (degree of freedom: 32, DF = 6). Abbreviations: Ara, arabinose; DM, degree of methyl esterification; Gal, galactose; Glc, glucose; Man, mannose; Rha, rhamnose; Xyl, xylose.

**Table 2:** Chemical characteristics of the polysaccharide fractions obtained during fractionation of EA-4.0 and EA-0.8 on DEAE-cellulose column (OH^−^).

Characteristic	EA-4.0						EA-0.8					
	0.01 NaCl	0.01p ^4^ NaCl	0.1 NaCl	0.2 NaCl	0.3 NaCl	0.2 NaOH	0.01 NaCl	0.01p ^4^ NaCl	0.1 NaCl	0.2 NaCl	0.3 NaCl	0.2 NaOH
Yield, % ^1^	12.33	19.97	11.04	29.50	5.82	1.68	11.48	13.94	16.39	34.01	7.78	1.10
Mw, kDa	254	323	88	100	102	n.d.	172	232	61	90	93	n.d.
PDI	4.67	3.73	4.72	2.28	2.21	n.d.	4.26	3.05	3.86	2.42	2.55	n.d.
Uronic acid, % ^2^	86.9	91.3	93.4	91.5	93.8	76.0	84.9	89.0	93.4	97.0	97.4	39.4
Protein, % ^3^	0.0	0.0	0.0	0.0	0.0	0.0	0.0	0.0	0.0	0.0	0.0	n.d.
Phenolic, % ^3^	0.5	0.5	0.5	0.9	0.6	4.1	1.1	1.5	0.4	0.3	0.3	n.d.
Rha, % ^2^	1.9	1.6	1.3	1.9	1.7	1.8	3.1	2.4	1.3	0.7	0.4	7.4
Ara, % ^2^	4.7	1.9	1.6	2.8	1.7	6.2	2.4	1.6	1.6	0.6	0.4	9.7
Xyl, % ^2^	0.4	0.6	0.6	0.4	0.2	1.2	1.0	0.8	0.6	0.5	0.7	5.9
Man, % ^2^	0.9	0.8	0.4	0.8	0.2	0.8	1.6	1.4	0.4	0.3	0.2	5.3
Glc, % ^2^	1.3	1.0	0.5	0.5	1.0	4.4	1.7	1.4	0.5	0.4	0.6	13.9
Gal, % ^2^	3.9	2.8	2.1	2.1	1.5	9.6	5.3	3.6	2.1	0.6	0.4	18.4
Total sugar, % ^3^	54.82	65.29	61.60	69.71	68.07	37.57	49.54	55.16	56.84	71.09	70.72	40.84
GalA/NM	6.61	10.49	14.06	10.78	15.05	3.17	5.62	8.07	14.06	31.79	37.02	0.65
Rha/GalA	0.022	0.018	0.014	0.020	0.018	0.024	0.037	0.026	0.014	0.007	0.005	0.187
Ara + Gal/Rha	4.57	2.93	2.92	2.61	1.93	8.77	2.45	2.20	2.92	1.79	1.62	3.81
GalA–Rha	84.97	89.68	92.07	89.63	92.09	74.20	81.75	86.63	92.07	96.29	96.92	32.05
2Rha + Ara + Gal	12.41	7.98	6.35	8.62	6.56	19.38	13.97	9.87	6.35	2.50	1.63	42.82
Ara/Xyl	10.70	3.02	2.55	6.60	8.10	5.10	2.51	2.08	2.55	1.10	0.53	1.63
Xyl/GalA	0.005	0.007	0.007	0.005	0.002	0.016	0.011	0.009	0.007	0.005	0.007	0.150

^1^ per weight of dry wt. ^2^ mol%. ^3^ wt%. ^4^ Pigmented fraction. Abbreviations: Ara, arabinose; Gal, galactose; Glc, glucose; Man, mannose; Mw, weight-average molecular weight; PDI, polydispersity index; n.d., not detected; Rha, rhamnose; Xyl, xylose.

**Table 3:** Chemical characteristics of the polysaccharide fractions (1–5 and residue) obtained by fractionated precipitation with ethanol of products of the enzymatic digestion of EA-4.0 and chemical characterization of the polysaccharide fractions obtained during fractionation of residue of enzymatic digestion on DEAE-cellulose column (OH^−^).

Characteristic	Enzymatic Digestion						Elution of Residue				
	EtOH-1	EtOH-2	EtOH-3	EtOH-4	EtOH-5	Residue	0.01 NaCl	0.1 NaCl	0.2 NaCl	0.3 NaCl	0.2 NaOH
Yield, % ^1^	20.03	1.70	1.53	4.11	2.36	53.37	5.52	35.42	11.56	3.97	4.02
Mw, kDa	3	n.d.	6	n.d.	n.d.	82	178	39	65	72	n.d.
PDI	1.39	n.d.	2.00	n.d.	n.d.	4.37	2.72	2.31	1.66	2.27	n.d.
Uronic acid,% ^2^	90.8	46.9	88.3	67.7	72.4	83.2	69.1	90.1	95.4	95.1	64.5
Protein, % ^3^	0.0	4.4	4.4	6.5	5.9	1.6	0.1	0.0	0.0	0.0	5.4
Phenolic, % ^3^	3.0	3.8	7.5	3.2	3.8	3.3	1.0	0.3	0.5	0.6	7.3
Rha, % ^2^	0.4	1.1	0.9	1.2	1.1	2.9	6.4	2.6	1.0	1.1	4.4
Ara, % ^2^	1.8	4.9	1.7	6.3	7.0	2.5	4.0	1.9	0.5	0.9	7.2
Xyl, % ^2^	2.5	7.1	2.2	10.5	8.7	3.1	1.4	1.2	1.0	1.0	2.0
Man, % ^2^	0.0	1.3	0.0	0.0	0.0	1.1	4.4	0.7	0.4	0.3	1.0
Glc, % ^2^	3.6	35.8	5.4	11.2	8.4	3.1	3.5	0.6	0.8	0.8	8.9
Gal, % ^2^	1.1	2.9	1.6	3.1	2.5	4.2	11.2	2.8	1.0	0.9	12.0
Total sugar, % ^3^	96.25	62.20	62.02	46.80	52.90	75.74	50.75	66.21	64.55	62.54	20.38
GalA/NM	9.81	0.88	7.58	2.10	2.62	4.93	2.23	9.14	20.51	19.37	1.82
Rha/GalA	0.004	0.023	0.010	0.018	0.015	0.035	0.093	0.028	0.010	0.011	0.069
Ara + Gal/Rha	7.28	7.27	3.67	7.81	8.93	2.28	2.38	1.86	1.54	1.65	4.33
GalA-Rha	90.36	45.82	87.45	66.50	71.33	80.22	62.66	87.59	94.36	94.05	60.05
2Rha + Ara + Gal	3.62	10.01	4.99	11.77	11.69	12.53	28.01	9.85	3.50	3.83	28.09
Ara/Xyl	0.71	0.69	0.77	0.60	0.81	0.80	2.87	1.56	0.52	0.84	3.70
Xyl/GalA	0.027	0.151	0.024	0.155	0.120	0.037	0.020	0.014	0.010	0.011	0.030

^1^ per weight of dry wt. ^2^ mol%. ^3^ wt.%. Abbreviations: Ara, arabinose; Gal, galactose; Glc, glucose; Man, mannose; Mw, weight-average molecular weight; PDI, polydispersity index; n.d., not detected; Rha, rhamnose; Xyl, xylose.

**Table 4:** Chemical characteristics of the polysaccharide fractions (1, 2 and residue) obtained by fractionated precipitation with ethanol of products of the enzymatic digestion of EA-0.8 and chemical characterization of the polysaccharide fractions obtained during fractionation of residue of enzymatic digestion on DEAE-cellulose column (OH^−^).

Characteristic	Enzymatic Digestion			Elution of Residue				
	EtOH-1	EtOH-2	Residue	0.01 NaCl	0.1 NaCl	0.2 NaCl	0.3 NaCl	0.2 NaOH
Yield, % ^1^	3.49	29.30	56.64	8.51	12.97	30.58	14.70	2.19
Mw, kDa	21	4	71	170	20	46	83	n.d.
PDI	5.36	1.47	4.51	4.08	2.17	1.91	1.76	n.d.
Uronic acid, % ^2^	75.2	80.9	85.8	60.2	94.8	96.0	98.5	70.4
Protein, % ^3^	3.2	0.0	0.2	1.3	0.0	0.0	0.0	2.7
Phenolic, % ^3^	5.8	1.9	2.2	2.1	0.2	0.3	0.4	5.8
Rha, % ^2^	4.2	0.6	2.3	8.5	0.8	1.0	0.4	3.6
Ara, % ^2^	3.0	1.5	1.7	5.6	2.0	0.8	0.3	5.1
Xyl, % ^2^	5.0	7.1	3.4	1.0	0.3	0.2	0.1	2.2
Man, % ^2^	0.4	0.1	1.1	6.1	0.2	0.7	0.0	1.4
Glc, % ^2^	8.0	7.9	2.4	4.6	0.3	0.3	0.2	7.1
Gal, % ^2^	4.2	1.8	3.2	14.0	1.7	1.1	0.5	10.2
Total sugar, % ^3^	70.49	68.58	77.22	54.52	65.93	79.39	78.49	23.48
GalA/NM	3.04	4.23	6.04	1.51	18.38	23.69	64.80	2.38
Rha/GalA	0.056	0.008	0.027	0.141	0.008	0.010	0.004	0.051
Ara + Gal/Rha	1.70	5.14	2.10	2.31	4.71	2.03	2.02	4.27
GalA–Rha	71.02	80.24	83.46	51.67	94.06	94.98	98.08	66.84
2Rha + Ara + Gal	15.61	4.64	9.60	36.64	5.23	3.91	1.65	22.43
Ara/Xyl	0.60	0.21	0.50	5.69	7.00	5.19	3.40	2.27
Xyl/GalA	0.066	0.088	0.040	0.016	0.003	0.002	0.001	0.032

^1^ per weight of dry wt. ^2^ mol%. ^3^ wt.%. Abbreviations: Ara, arabinose; Gal, galactose; Glc, glucose; Man, mannose; Mw, weight-average molecular weight; PDI, polydispersity index; n.d., not detected; Rha, rhamnose; Xyl, xylose.

**Table 5:** The DPPH scavenging activity of fireweed pectins in comparison with commercial apple pectin (AP) and trolox.

Pectin/Standard	IC_50_ (mg/mL)
EA-4.0	0.050 ± 0.003 ^a^
EA-0.8	0.109 ± 0.003 ^b^
AP (control)	1.961 ± 0.363 ^c^
Trolox (standard)	0.005 ± 0.000 ^d^

Data are presented as the mean ± SD of three independent experiments. Different lowercase letters (a, b, c, d) indicate the significant differences (*p* < 0.05, LSD test).

**Table 6:** The Pearson correlation coefficients between DPPH scavenging activity (in %) with the physicochemical characteristics of pectins and fractions (*n* = 32).

Second Variable **	R	*p*	Second Variable	R	*p*
GalA	−0.55	0.001	Mw *	−0.40	0.053
GalA–Rha	−0.53	0.002	Carbohydrate	−0.18	0.338
GalA/NM	−0.47	0.006	Man	−0.06	0.737
Ara/Xyl	−0.44	0.011	Rha	0.18	0.325
(Ara + Gal)/Rha	0.36	0.042	Rha/GalA	0.20	0.267
Ara	0.44	0.011	PDI *	0.21	0.313
Glc	0.50	0.004	Gal	0.26	0.146
Xyl/GalA	0.55	0.001	2Rha + Ara + Gal	0.30	0.096
Xyl	0.58	0.000			
Protein	0.72	0.000			
Phenolic	0.93	0.000			

* *n* = 24. ** Significantly correlated variables are grouped in this column.

**Table 7:** The multiple regression analysis for percentage of DPPH scavenging activity with the characteristics of pectins and fractions.

Variable		*β*	Standard Error of *β*	Parameter Estimate	Standard Error	*p* Value
Dependent	Independent					
DPPH scavenging activity	Phenolics	0.85	0.07	2.27	0.17	0.000
	Xyl/GalA	0.20	0.07	280.73	91.11	0.004

Regression results: R^2^ = 0.897, adjusted R^2^ = 0.890, F_2.29_ = 125.970, *p* < 0.000, Standard estimate error = 19.135.

## Data Availability

The data that support the findings of this study are available from the corresponding author upon reasonable request.

## Sample Availability

Samples of the compounds are available from the authors.

## Abbreviations

Ara	Arabinose
DM	Degree of methyl esterification
Gal	Galactose
Glc	Glucose
Man	Mannose
Rha	Rhamnose
Xyl	Xylose

## References

[1] Adamczak A., Dreger M., Seidler-Łożykowska K., Wielgus K. (2019). Fireweed (*Epilobium angustifolium* L.): Botany, phytochemistry and traditional uses. A review. Herba Pol..

[2] Lasinskas M., Jariene E., Vaitkeviciene N., Hallmann E., Najman K. (2020). Effect of Different Durations of Solid-Phase Fermentation for Fireweed (*Chamerion angustifolium* (L.) Holub) Leaves on the Content of Polyphenols and Antioxidant Activity In Vitro. Molecules.

[3] Deng L., Zong W., Tao X., Liu S., Feng Z., Lin Y., Liao Z., Chen M. (2019). Evaluation of the therapeutic effect against benign prostatic hyperplasia and the active constituents from *Epilobium angustifolium* L.. J. Ethnopharmacol..

[4] Schepetkin I.A., Ramstead A.G., Kirpotina L.N., Voyich J.M., Jutila M.A., Quinn M.T. (2016). Therapeutic Potential of Polyphenols from *Epilobium angustifolium* (Fireweed). Phytother. Res..

[5] Vitalone A., Allkanjari O. (2018). *Epilobium* spp: Pharmacology and Phytochemistry. Phytother. Res..

[6] Yang Y., Anderson C.T. (2020). Biosynthesis, Localisation, and Function of Pectins in Plants. Pectin: Technological and Physiological Properties.

[7] Voragen A.G.J., Coenen G.-J., Verhoef R.P., Schols H.A. (2009). Pectin, a versatile polysaccharide present in plant cell walls. Struct. Chem..

[8] Minzanova S.T., Mironov V.F., Arkhipova D.M., Khabibullina A.V., Mironova L.G., Zakirova Y.M., Milyukov V.A. (2018). Biological Activity and Pharmacological Application of Pectic Polysaccharides: A Review. Polymers.

[9] Tan M., Chang S., Liu J., Li H., Xu P., Wang P., Wang X., Zhao M., Zhao B., Wang L. (2020). Physicochemical Properties, Antioxidant and Antidiabetic Activities of Polysaccharides from Quinoa (*Chenopodium quinoa* Willd.) Seeds. Molecules.

[10] Wu D.-T., Liu W., Han Q.-H., Wang P., Xiang X.-R., Ding Y., Zhao L., Zhang Q., Li S.-Q., Qin W. (2019). Extraction Optimization, Structural Characterization, and Antioxidant Activities of Polysaccharides from Cassia Seed (*Cassia obtusifolia*). Molecules.

[11] He Z., Zhu Y., Bao X., Zhang L., Li N., Jiang G., Peng Q. (2019). Optimization of Alkali Extraction and Properties of Polysaccharides from *Ziziphus jujuba* cv. Residue. Molecules.

[12] Patova O., Smirnov V., Golovchenko V., Vityazev F., Shashkov A., Popov S. (2019). Structural, rheological and antioxidant properties of pectins from *Equisetum arvense* L. and *Equisetum sylvaticum* L.. Carbohydr. Polym..

[13] Zeng F., Chen W., He P., Zhan Q., Wang Q., Wu H., Zhang M. (2020). Structural characterization of polysaccharides with potential antioxidant and immunomodulatory activities from Chinese water chestnut peels. Carbohydr. Polym..

[14] Yeung Y.K., Kang Y.-R., So B.R., Jung S.K., Chang Y.H. (2021). Structural, antioxidant, prebiotic and anti-inflammatory properties of pectic oligosaccharides hydrolyzed from okra pectin by Fenton reaction. Food Hydrocoll..

[15] Zhang T., Shuai M., Ma P., Huang J., Sun C., Yao X., Chen Z., Min X., Yan S. (2020). Purification, chemical analysis and antioxidative activity of polysaccharides from pH-modified citrus pectin after dialyzation. LWT.

[16] Ma X., Yu J., Jing J., Zhao Q., Ren L., Hu Z. (2021). Optimization of sunflower head pectin extraction by ammonium oxalate and the effect of drying conditions on properties. Sci. Rep..

[17] Ning X., Liu Y., Jia M., Wang Q., Sun Z., Ji L., Mayo K.H., Zhou Y., Sun L. (2021). Pectic polysaccharides from Radix Sophorae Tonkinensis exhibit significant antioxidant effects. Carbohydr. Polym..

[18] Wang W., Ma X., Jiang P., Hu L., Zhi Z., Chen J., Ding T., Ye X., Liu D. (2016). Characterization of pectin from grapefruit peel: A comparison of ultrasound-assisted and conventional heating extractions. Food Hydrocoll..

[19] Wang X., Lü X. (2014). Characterization of pectic polysaccharides extracted from apple pomace by hot-compressed water. Carbohydr. Polym..

[20] Wang J., Hu S., Nie S., Yu Q., Xie M. (2016). Reviews on Mechanisms of In Vitro Antioxidant Activity of Polysaccharides. Oxid. Med. Cell. Longev..

[21] Donchenko L.V., Limareva N.S., Belousova A.I., Koss A.N. (2021). Pectin-containing antioxidant drink based on extracts of grape pomace and Chamerion. IOP Conf. Ser. Earth Environ. Sci..

[22] Olawuyi I.F., Lee W.Y. (2021). Structural characterization, functional properties and antioxidant activities of polysaccharide extract obtained from okra leaves (*Abelmoschus esculentus*). Food Chem..

[23] Ezzati S., Ayaseh A., Ghanbarzadeh B., Heshmati M.K. (2020). Pectin from sunflower by-product: Optimization of ultrasound-assisted extraction, characterization, and functional analysis. Int. J. Biol. Macromol..

[24] Chen Q., Xue G., Ni Q., Wang Y., Gao Q., Zhang Y., Xu G. (2020). Physicochemical and rheological characterization of pectin-rich polysaccharides from *Gardenia jasminoides* J. Ellis flower. Food Sci. Nutr..

[25] Hosseini S.S., Khodaiyan F., Kazemi M., Najari Z. (2019). Optimization and characterization of pectin extracted from sour orange peel by ultrasound assisted method. Int. J. Biol. Macromol..

[26] Buathongjan C., Israkarn K., Sangwan W., Outrequin T., Gamonpilas C., Methacanon P. (2020). Studies on chemical composition, rheological and antioxidant properties of pectin isolated from Riang (*Parkia timoriana* (DC.) Merr.) pod. Int. J. Biol. Macromol..

[27] Chen Y., Wang Y., Xu L., Jia Y., Xue Z., Zhang M., Phisalaphong M., Chen H. (2020). Ultrasound-assisted modified pectin from unripe fruit pomace of raspberry (*Rubus chingii* Hu): Structural characterization and antioxidant activities. LWT.

[28] Hosseini S., Parastouei K., Khodaiyan F. (2020). Simultaneous extraction optimization and characterization of pectin and phenolics from sour cherry pomace. Int. J. Biol. Macromol..

[29] Kazemi M., Khodaiyan F., Hosseini S.S. (2019). Utilization of food processing wastes of eggplant as a high potential pectin source and characterization of extracted pectin. Food Chem..

[30] Golbargi F., Gharibzahedi S.M.T., Zoghi A., Mohammadi M., Hashemifesharaki R. (2021). Microwave-assisted extraction of arabinan-rich pectic polysaccharides from melon peels: Optimization, purification, bioactivity, and techno-functionality. Carbohydr. Polym..

[31] La Cava E.L., Gerbino E., Sgroppo S.C., Gómez-Zavaglia A. (2018). Characterization of Pectins Extracted from Different Varieties of Pink/Red and White Grapefruits [*Citrus Paradisi* (Macf.)] by Thermal Treatment and Thermosonication. J. Food Sci..

[32] Lefsih K., Delattre C., Pierre G., Michaud P., Aminabhavi T., Dahmoune F., Madani K. (2016). Extraction, characterization and gelling behavior enhancement of pectins from the cladodes of *Opuntia ficus* indica. Int. J. Biol. Macromol..

[33] Qin Z., Liu H.-M., Lv T.-T., Wang X.-D. (2020). Structure, rheological, thermal and antioxidant properties of cell wall polysaccharides from Chinese quince fruits. Int. J. Biol. Macromol..

[34] Wang W., Liu J. (2020). Efficient extraction, antioxidant activities and anti-inflammation of polysaccharides from *Notopterygium franchetii* Boiss. Carbohydr. Polym..

[35] Ke J., Jiang G., Shen G., Wu H., Liu Y., Zhang Z. (2020). Optimization, characterization and rheological behavior study of pectin extracted from chayote (*Sechium edule*) using ultrasound assisted method. Int. J. Biol. Macromol..

[36] Rahmani Z., Khodaiyan F., Kazemi M., Sharifan A. (2020). Optimization of microwave-assisted extraction and structural characterization of pectin from sweet lemon peel. Int. J. Biol. Macromol..

[37] Yapo B.M. (2011). Pectic substances: From simple pectic polysaccharides to complex pectins—A new hypothetical model. Carbohydr. Polym..

[38] Garna H., Mabon N., Wathelet B., Paquot M. (2004). New Method for a Two-Step Hydrolysis and Chromatographic Analysis of Pectin Neutral Sugar Chains. J. Agric. Food Chem..

[39] Zhang T., Liu H., Bai X., Liu P., Yang Y., Huang J., Zhou L., Min X. (2020). Fractionation and antioxidant activities of the water-soluble polysaccharides from Lonicera japonica Thunb. Int. J. Biol. Macromol..

[40] Li M., Li T., Hu X., Ren G., Zhang H., Wang Z., Teng Z., Wu R., Wu J. (2021). Structural, rheological properties and antioxidant activities of polysaccharides from mulberry fruits (*Murus alba* L.) based on different extraction techniques with superfine grinding pretreatment. Int. J. Biol. Macromol..

[41] Xia Y.-G., Huang Y.-X., Liang J., Kuang H.-X. (2020). Comparable studies of two polysaccharides from leaves of *Acanthopanax senticosus*: Structure and antioxidation. Int. J. Biol. Macromol..

[42] Chen X., Qi Y., Zhu C., Wang Q. (2019). Effect of ultrasound on the properties and antioxidant activity of hawthorn pectin. Int. J. Biol. Macromol..

[43] Wang L., Li L., Gao J., Huang J., Yang Y., Xu Y., Liu S., Yu W. (2021). Characterization, antioxidant and immunomodulatory effects of selenized polysaccharides from dandelion roots. Carbohydr. Polym..

[44] Wikiera A., Grabacka M., Byczyński Ł., Stodolak B., Mika M. (2021). Enzymatically Extracted Apple Pectin Possesses Antioxidant and Antitumor Activity. Molecules.

[45] Yi Y., Lamikanra O., Sun J., Wang L.-M., Min T., Wang H.-X. (2018). Activity diversity structure-activity relationship of polysaccharides from lotus root varieties. Carbohydr. Polym..

[46] Li Z., Nie K., Wang Z., Luo D. (2016). Quantitative Structure Activity Relationship Models for the Antioxidant Activity of Polysaccharides. PLoS ONE.

[47] Lo T.C.-T., Chang C.A., Chiu K.-H., Tsay P.-K., Jen J.-F. (2011). Correlation evaluation of antioxidant properties on the monosaccharide components and glycosyl linkages of polysaccharide with different measuring methods. Carbohydr. Polym..

[48] Shafie M.H., Gan C.-Y. (2020). Could choline chloride-citric acid monohydrate molar ratio in deep eutectic solvent affect structural, functional and antioxidant properties of pectin?. Int. J. Biol. Macromol..

[49] Patova O., Golovchenko V.V., Vityazev F.V., Burkov A., Belyi V.A., Kuznetsov S.N., Litvinets S., Martinson E.A. (2017). Physicochemical and rheological properties of gelling pectin from Sosnowskyi’s hogweed (*Heracleum sosnowskyi*) obtained using different pretreatment conditions. Food Hydrocoll..

[50] Dubois M., Gilles K.A., Hamilton J.K., Rebers P.A., Smith F. (1956). Colorimetric Method for Determination of Sugars and Related Substances. Anal. Chem..

[51] Liu X., Renard C.M., Rolland-Sabaté A., Bureau S., Le Bourvellec C. (2021). Modification of apple, beet and kiwifruit cell walls by boiling in acid conditions: Common and specific responses. Food Hydrocoll..

[52] Usov A.I., Bilan M.I., Klochkova N.G. (1995). Polysaccharides of Algae. 48. Polysaccharide Composition of Several Calcareous Red Algae: Isolation of Alginate from *Corallina pilulifera* P. et R. (Rhodophyta, Corallinaceae). Bot. Mar..

[53] Bradford M.M. (1976). A rapid and sensitive method for the quantitation of microgram quantities of protein utilizing the principle of protein-dye binding. Anal. Biochem..

[54] Ruviaro A.R., Barbosa P.D.P.M., Martins I.M., de Ávila A.R.A., Nakajima V.M., Dos Prazeres A.R., Macedo J.A., Macedo G.A. (2020). Flavanones biotransformation of citrus by-products improves antioxidant and ACE inhibitory activities in vitro. Food Biosci..

[55] Golovchenko V.V., Naranmandakh S., Ganbaatar J., Prilepskii A.Y., Burygin G.L., Chizhov A.O., Shashkov A.S. (2020). Structural investigation and comparative cytotoxic activity of water-soluble polysaccharides from fruit bodies of the medicinal fungus quinine conk. Phytochemistry.

[56] Wood P., Siddiqui I. (1971). Determination of methanol and its application to measurement of pectin ester content and pectin methyl esterase activity. Anal. Biochem..

[57] Houben K., Jolie R.P., Fraeye I., Van Loey A., Hendrickx M.E. (2011). Comparative study of the cell wall composition of broccoli, carrot, and tomato: Structural characterization of the extractable pectins and hemicelluloses. Carbohydr. Res..

[58] Nelson N. (1944). A photometric adaptation of the Somogyi method for the determination of glucose. J. Biol. Chem..

[59] Shogren R.L., Biswas A. (2013). Preparation of starch–sodium lignosulfonate graft copolymers via laccase catalysis and characterization of antioxidant activity. Carbohydr. Polym..

[60] Quick K.L., Hardt J.I., Dugan L.L. (2000). Rapid microplate assay for superoxide scavenging efficiency. J. Neurosci. Methods.

